# Supplementary material for: Blue Titania: The Outcome of Defects, Crystalline-Disordered Core-Shell Structure, and Hydrophilicity Change
Source: Nanomaterials (Basel). 2022 Apr 28;12(9):1501. doi: 10.3390/nano12091501 (PMC9104741; doi:10.3390/nano12091501)
Supplement: Supplementary file 1 [file nanomaterials-12-01501-s001.zip › nanomaterials-1646359-supplementary.pdf]

nanomaterials

Supplementary Materials

Blue Titania: The Outcome of Defects, Crystalline-Disordered Core-Shell Structure, and Hydrophilicity Change

Sergio A. Sabinas-Hernández [1,*], Justo Miguel Gracia Jiménez [1], Nicolás Rutilo Silva González [1], María P. Elizalde-González [2], Ulises Salazar-Kuri [1] and Samuel Tehuacanero-Cuapa [3]

[1] Instituto de Física, Benemérita Universidad Autónoma de Puebla, Av. San Claudio y Blvd. 18 Sur, Col. San Manuel, Ciudad Universitaria, Apartado postal J-48, Puebla 72570, Mexico; gracia@ifuap.buap.mx (J.M.G.J.); silva@ifuap.buap.mx (N.R.S.G.); usalazar@ifuap.buap.mx (U.S.-K.)
[2] Centro de Química, Instituto de Ciencias, Benemérita Universidad Autónoma de Puebla, Ciudad Universitaria, Edif. IC7, Puebla 72570, Mexico; maria.elizalde@correo.buap.mx (M.P.E.G.)
[3] Instituto de Física, Universidad Nacional Autónoma de México, Circuito de la Investigación s/n, Ciudad Universitaria, Ciudad de México 04510, Mexico; samueltc@fisica.unam.mx
* Correspondence: ssabinas@ifuap.buap.mx

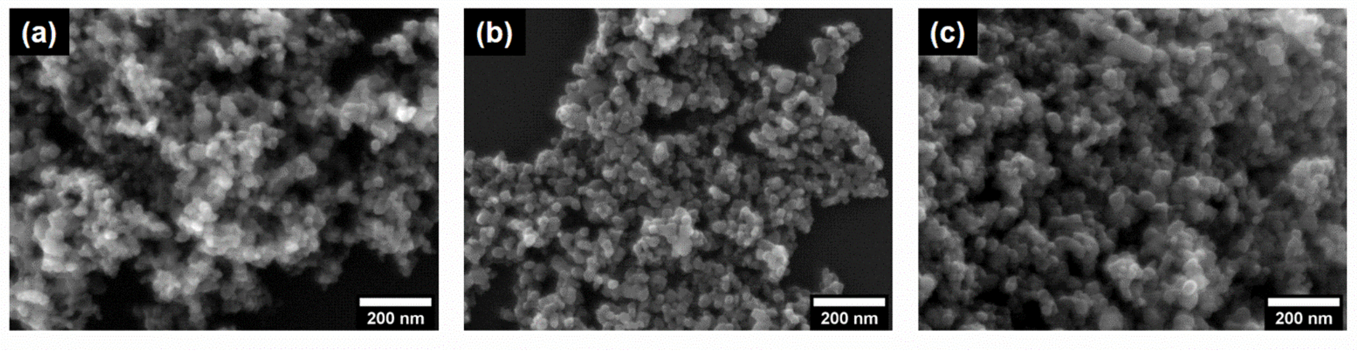

Figure S1. SEM images of (**a**) P25, (**b**) BT(3.88), and (**c**) BT(6.64).

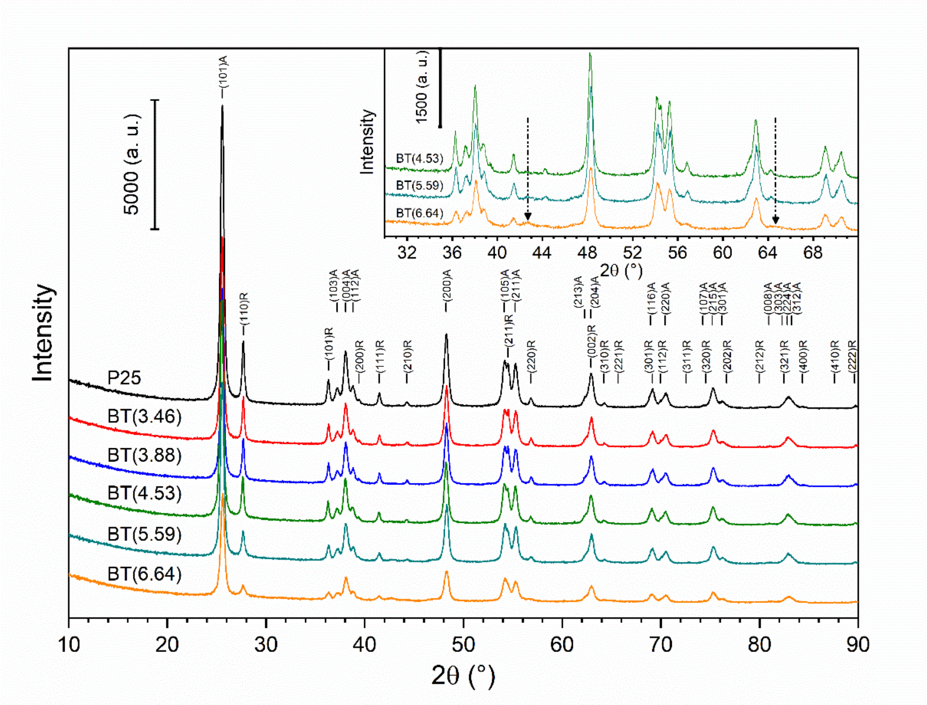

Figure S2. Diffraction patterns of the prepared samples. Inset shows the presence of two small broad unidentified peaks at ~42.7° and ~64.8°.

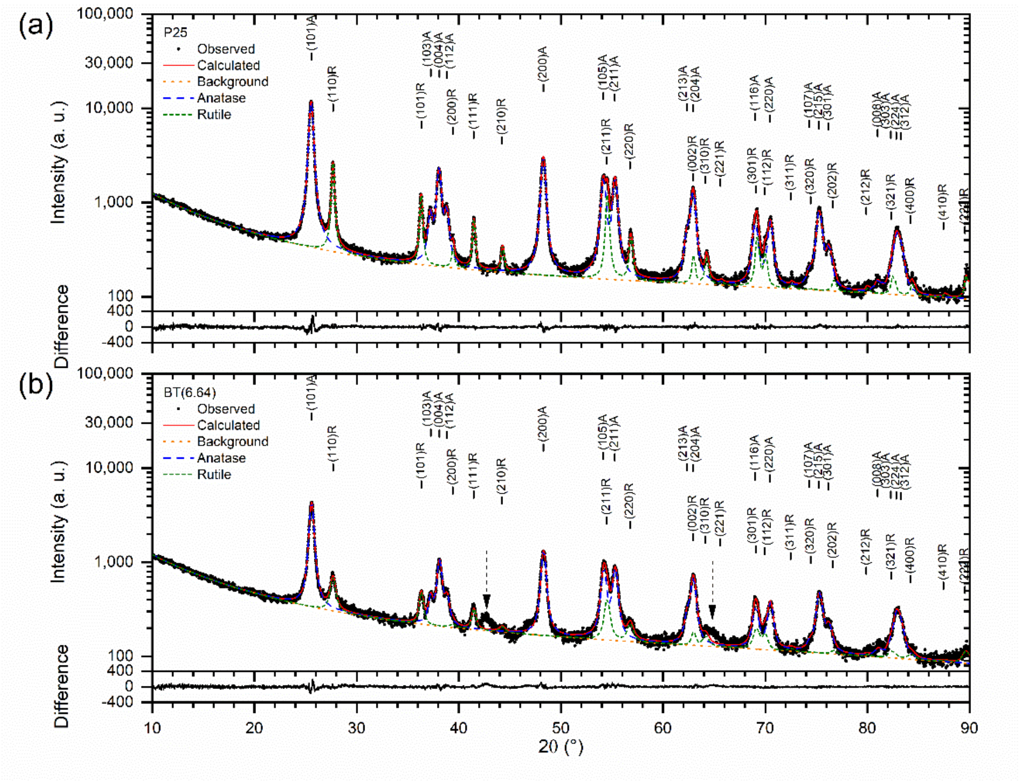

Figure S3. Refinement of (**a**) P25 and (**b**) BT(6.64) samples in logarithmic scale. Difference between observed and calculated data in linear scale is shown at the bottom of each diffractogram.

Table S1. Lattice parameters of the samples and R values obtained after Rietveld refinement.

**Sample**	**Anatase**			**Rutile**			R_{wp}	χ^2
	**a (Å)**	**c (Å)**	**c/a**	**a (Å)**	**c (Å)**	**c/a**	**(%)**	$(R_{wp}/R_{exp})^2$
P25	3.7928(1)	9.5248(3)	2.5113(1)	4.6029(2)	2.9649(1)	0.6441(1)	4.67	1.00
BT(3.46)	3.7901(9)	9.517(2)	2.5111(2)	4.600(1)	2.9622(7)	0.6440(7)	5.10	1.04
BT(3.88)	3.7901(9)	9.517(2)	2.5109(2)	4.600(1)	2.9621(7)	0.6440(7)	4.97	1.00
BT(4.53)	3.7912(1)	9.5186(3)	2.5107(1)	4.6018(2)	2.9634(2)	0.6440(1)	4.86	1.01
BT(5.59)	3.787(1)	9.507(3)	2.5104(2)	4.598(1)	2.9600(9)	0.6437(9)	5.43	1.21
BT(6.64)	3.7945(2)	9.5243(5)	2.5100(1)	4.6109(6)	2.9666(5)	0.6434(5)	6.35	1.41

Table S2. Atomic coordinates and thermal displacement parameter obtained from Rietveld refinement.

**Sample**	**Anatase**			**Rutile**		
	**O Position (z)**	U_{equiv}**(Ti) (Å²)**	U_{equiv}**(O) (Å²)**	**O Position (x)**	U_{equiv}**(Ti) (Å²)**	U_{equiv}**(O) (Å²)**
P25	0.2081(4)	0.0142(2)	0.0227(5)	0.3068(5)	0.0162(8)	0.024(1)
BT(3.46)	0.2093(2)	0.0004(3)	0.0	0.3041(7)	0.004(1)	0.011(2)
BT(3.88)	0.2098(2)	0.0	0.0023(5)	0.3046(6)	0.0	0.003(1)
BT(4.53)	0.2087(2)	0.0050(3)	0.0107(5)	0.3028(7)	0.004(1)	0.014(2)
BT(5.59)	0.2103(2)	0.0	0.0018(6)	0.3005(9)	0.0	0.003(2)
BT(6.64)	0.2093(2)	0.0	0.0036(9)	0.297(1)	0.0	0.0

Table S3. Crystal size along different crystallographic directions and mean crystallite size. These sizes correspond to a volume weighted mean sphere diameter. The column length size can be obtained multiplying the size by ¾.

Sample	Anatase			Rutile		
	<1 0 0> (nm)	<0 0 1> (nm)	Mean Sphere Diameter (nm)	<1 0 0> (nm)	<0 0 1> (nm)	Mean Sphere Diameter (nm)
P25	23.7(1)	21.3(2)	22.8	34.8(4)	40(1)	36.3
BT(3.46)	24.7(2)*	--	24.7	38.8(5)	46(2)	40.8
BT(3.88)	24.5(2)	21.5(2)	23.4	36.8(5)	47(0)	39.6
BT(4.53)	24.6(2)	22.0(2)	23.6	32.7(4)	46(2)	35.9
BT(5.59)	23.3(1)	20.1(2)	22.1	23.0(4)	46(4)	26.9
BT(6.64)	20.4(2)	18.8(3)	19.8	16.1(5)	31(4)	18.7

* Isotropic crystal shape for anatase was assumed for the BT(3.46) sample.

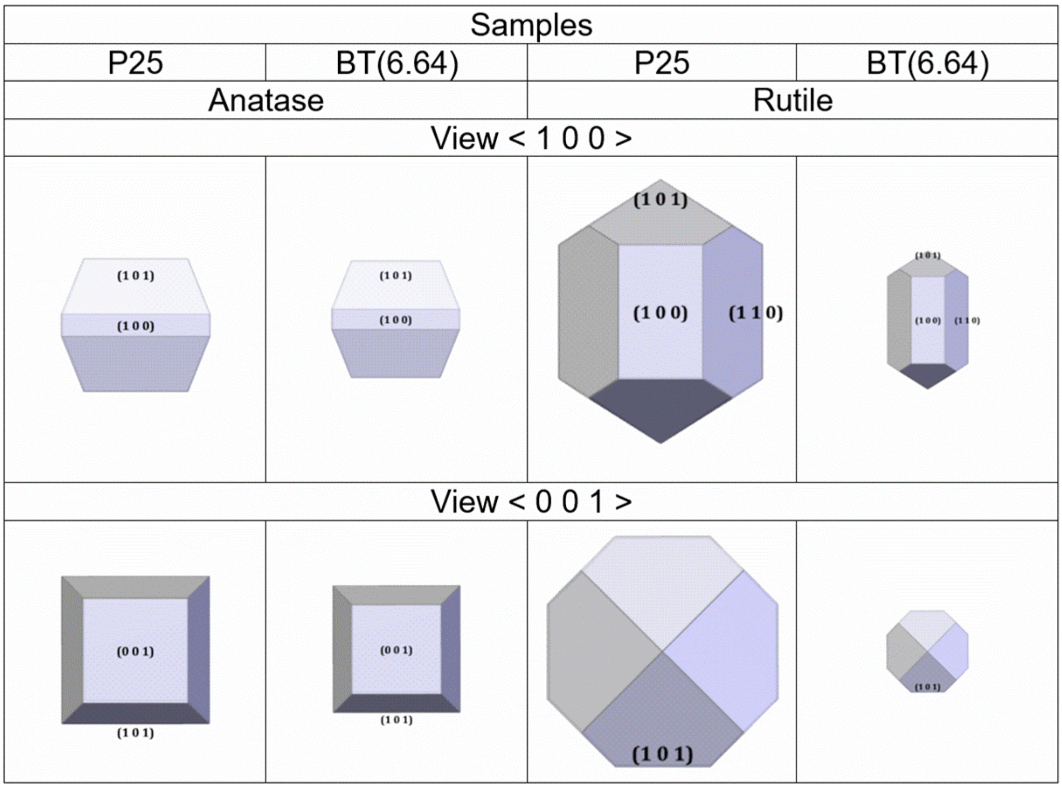

Figure S4. Crystallite models of anatase and rutile viewed from different directions, constructed considering only 3 low index planes with the minor surface energy. Models are not exactly to scale. Sizes along the <100> and <001> crystallographic directions are shown in Table S3.

Table S4. Percent of surface planes for anatase and rutile in two samples considering only 3 low index planes.

Phase	Plane	P25	BT(6.64)
		Area (%)	
Anatase	(101)	63.5	63.3
	(100)	13.7	14.5
	(001)	22.8	22.2
Rutile	(110)	28.9	34.0
	(100)	25.3	31.3
	(101)	45.8	34.7

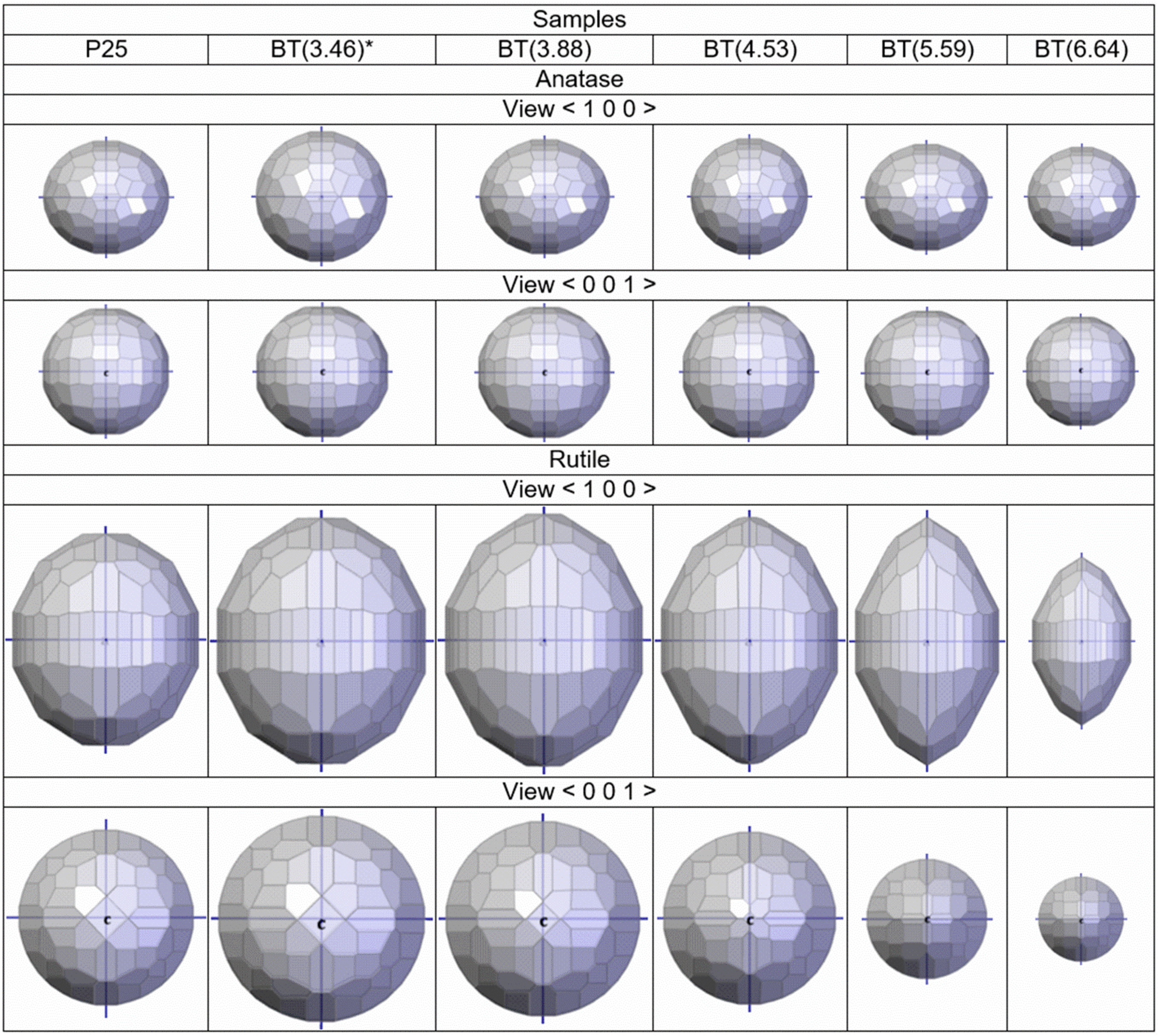

Figure S5. Crystallite models of anatase and rutile viewed from different directions, axis referred to the center of the crystallite. Models are not exactly to scale. Sizes along <100> and <001> crystallographic directions are shown in Table S3. *Isotropic crystal shape for anatase phase was assumed for BT(3.46).

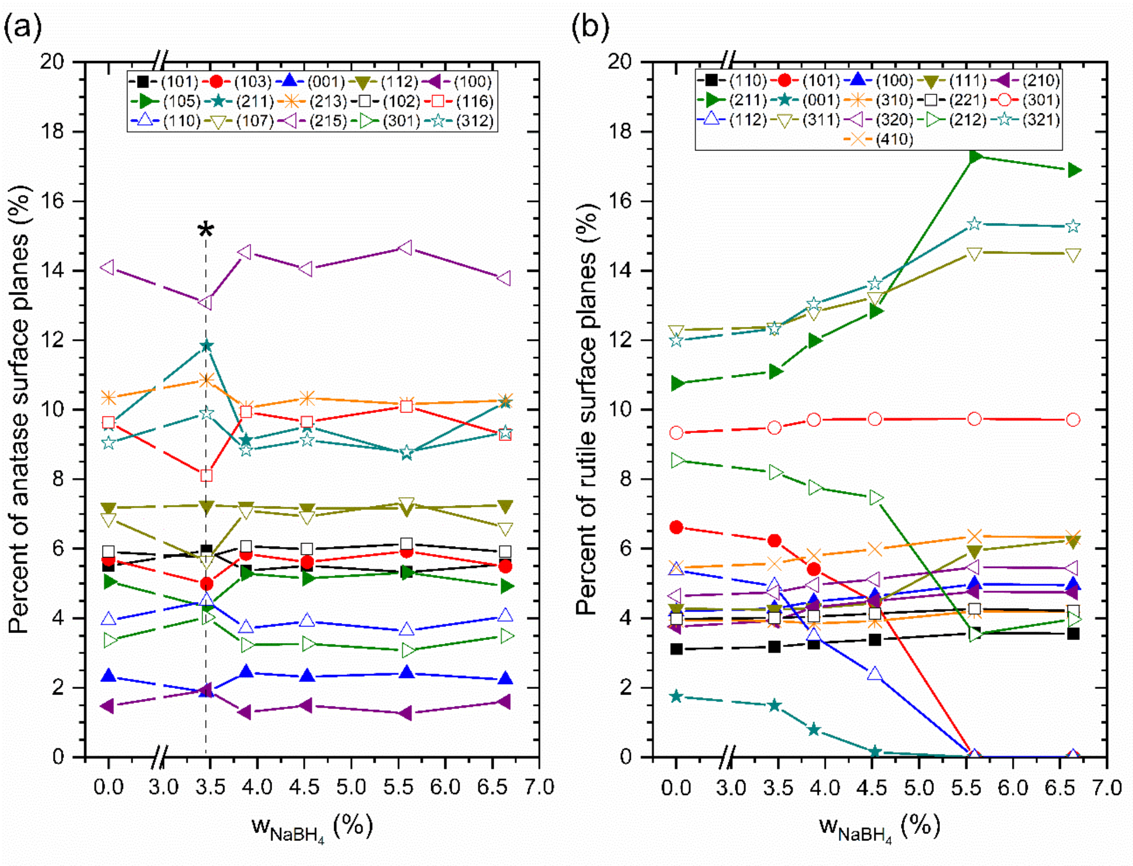

Figure S6. Area percent of surface planes for (**a**) anatase and (**b**) rutile in the samples. * Isotropic crystal shape for anatase was assumed in the BT(3.46) sample. It is indicated with a vertical dashed line in (**a**), therefore, it disagrees with the trend.

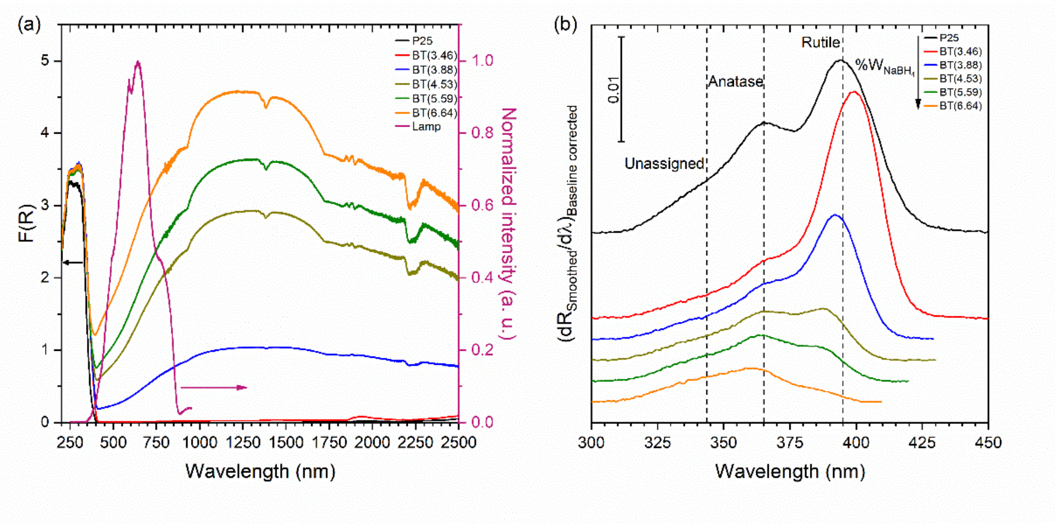

Figure S7. (**a**) Kubelka Munk function of all samples in the UV-Vis-NIR region and the emission spectrum of the lamp used in the photocatalytic experiments (**b**) Derivative of diffuse reflectance spectra of the samples. Dashed lines indicate the position of local maxima found in the P25 sample by deconvolution of bands (not shown).

Table S5. Chromaticity coordinates and chromatic parameters of samples in the CIE L*C*h* color system.

Sample	CIE 1931		CIE L*C*h* Space		
	x	y	Lightness (L*)	Chroma (C*)	Hue (h°)
P25	0.313	0.330	100	0.53	116.99
BT(3.46)	0.311	0.328	95	0.73	226.95
BT(3.88)	0.296	0.317	74	6.45	247.56
BT(4.53)	0.286	0.308	59	8.81	254.44
BT(5.59)	0.284	0.306	55	9.06	255.92
BT(6.64)	0.284	0.305	48	8.31	259.11

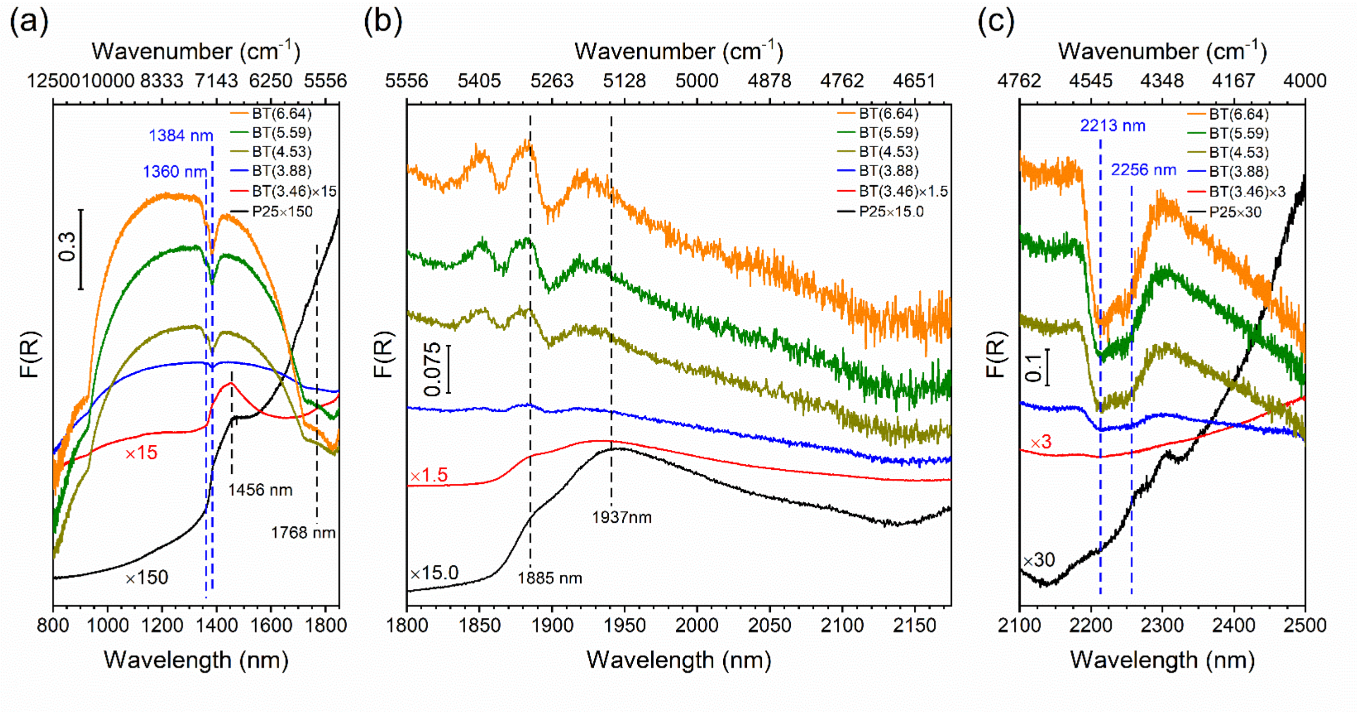

Figure S8. Sections of the near-infrared spectra of the samples: (**a**) 800 to 1850 nm, (**b**) 1800 to 2175 nm, and (**c**) 2100 to 2500 nm. Ordinate axes of all graphs were shifted for clarity.

Table S6. Specific surface area S_{BET} and total pore volume V_T.

Sample	SBET (m^2g^{-1})	VT (cm^3g^{-1})
P25	50	0.4133
BT(3.46)	59	0.4472
BT(3.88)	46	0.3992
BT(4.53)	48	0.4156
BT(5.59)	59	0.5075
BT(6.64)	56	0.5207

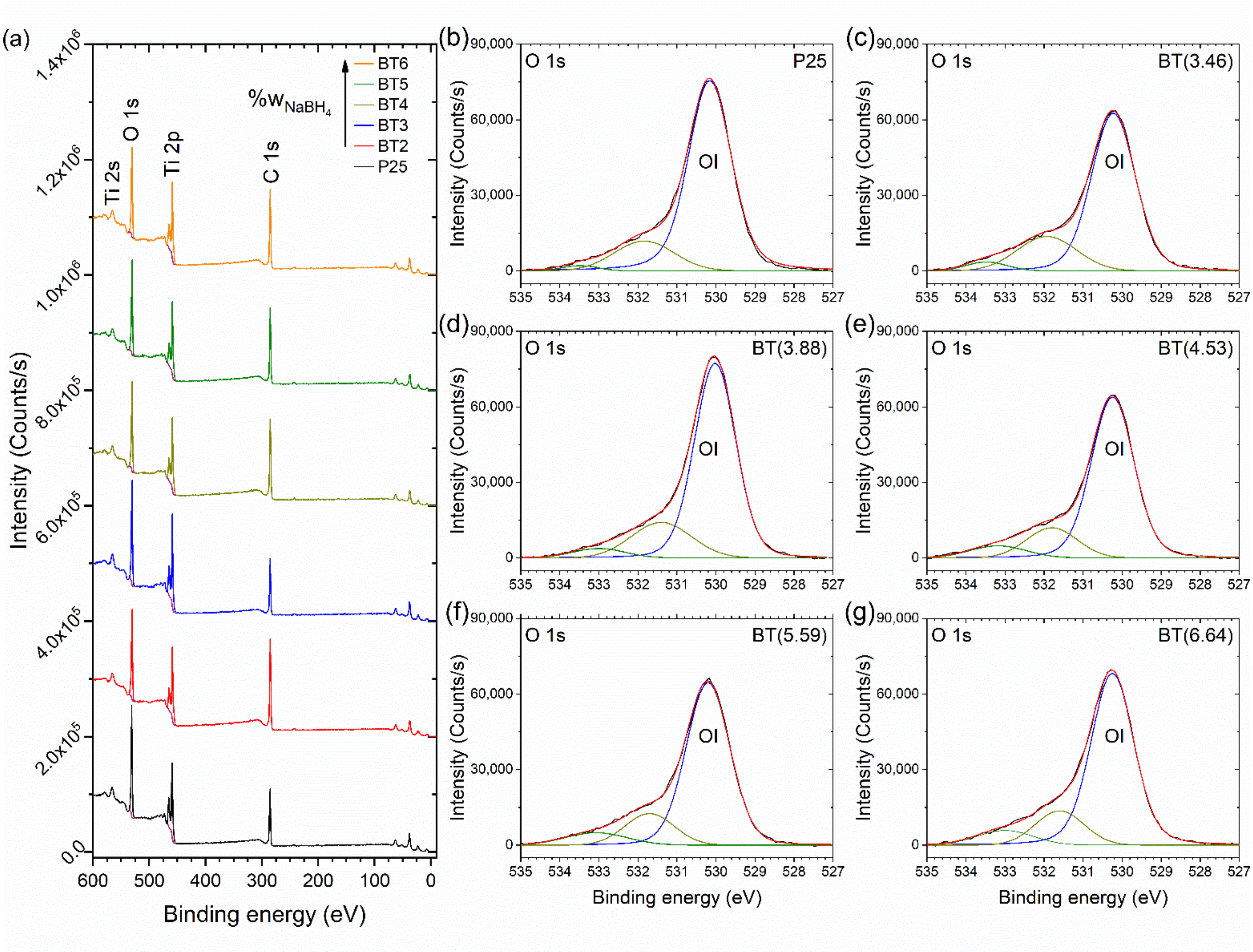

Figure S9. (**a**) Survey spectra and (**b**–**g**) XPS deconvoluted spectra of the O 1s-peak including the experimental curve and the theoretical fitted curve based on structural and nonstructural oxygen of the studied samples. A baseline correction of the Shirley type was applied in all cases.

Table S7. Dye removal percentage by 2 h of adsorption and 6 h photocatalysis of all samples, and the removal by 6 h of photolysis.

Sample	Removal of Dye Concentration (%) in the Solution by:		
	Photolysis	Adsorption	Photocatalysis
-	65 ± 5	-	-
P25	-	0	97 ± 5 *
BT(3.46)	-	0	89 ± 6
BT(3.88)	-	4 ± 15	95 ± 7
BT(4.53)	-	7 ± 9	88 ± 5
BT(5.59)	-	15 ± 9	84 ± 5
BT(6.64)	-	19 ± 9	76 ± 6

* This value was estimated from the kinetic model to be comparable with all samples.
